# Variations in the Rate of Infestations of Dogs with Zoonotic Nematodes and the Contamination of Soil in Different Environments

**DOI:** 10.3390/ijerph14091003

**Published:** 2017-09-01

**Authors:** Maria Bernadeta Studzińska, Marta Demkowska-Kutrzepa, Anna Borecka, Michał Meisner, Krzysztof Tomczuk, Monika Roczeń-Karczmarz, Teresa Kłapeć, Zahrai Abbass, Alicja Cholewa

**Affiliations:** 1 Department of Parasitology and Invasive Diseases, Faculty of Veterinary Medicine, University of Life Sciences in Lublin, Akademicka, 12, 20-033 Lublin, Poland; marta.demkowska@up.lublin.pl (M.D.-K.); krzysztof.tomczuk@up.lublin.pl (K.T.); monika.roczenkarczmarz@up.lublin.pl (M.R.-K.); 2 Laboratory of Parasitology, Military Institute of Hygiene and Epidemiology, Kozielska, 4, 01-163 Warsaw, Poland; aborecka1@tlen.pl; 3 Department of Psychology, Faculty of Social Sciences, The John Paul II Catholic University of Lublin, Al. Racławickie, 14, 20-950 Lublin, Poland; miszkasds@wp.pl; 4 Department of Biological Hazard and Parasitology, Institute of Rural Health in Lublin, Jaczewskiego, 2, 20-090 Lublin, Poland; teresaklapec@op.pl (T.K.); gchol@wp.pl (A.C.); 5 Department of Microbiology, Faculty of Medicine, Al Muthanna University, Samawa 66007, Iraq; zahraaabbas66@gmail.com

**Keywords:** *Toxocara canis*, Ancylostomatidae, dog, environment, zoonosis, LAMP

## Abstract

Companion animals are an important aspect in human life. However, they may also be considered a source of pathogens. An example of zoonotic parasitoses is toxocarosis or cutaneous larva migrans (CLM). The aim of the study was to detect zoonotic nematodes of dogs living in different areas and the intensity of contamination in parasite polluted environments that are hazardous to human health. The fecal samples were examined using standard flotation and decantation methods as well as McMaster’s quantitative technique. The soil samples in urban and rural areas were examined using a modified flotation method as described by Quinn et al. Statistical analyses were performed by IBM SPSS Statistics Version 23. The overall prevalence of parasites in dogs was 38%, 17.02% and 56.60% from urban and rural areas, respectively. The percentage values of nematodes important for human health (*Toxocara canis*, Ancylostomatidae, *Trichuris vulpis*) remained at the same level (16%). The infected dogs were dominated by a single parasite species, the main was *T. canis* (28.95%). In total, 54.30% of the soil samples were contaminated with parasite eggs. The contamination of urban and rural sandpits was 40% and 60%, respectively. The molecular examinations of soil samples using LAMP (loop-mediated isothermal amplification) confirmed the presence of nematode eggs of the species *T. canis* in all samples previously classified as positive

## 1. Introduction

Companion animals, especially cats and dogs, play a very important role in societies worldwide. These animals exert a beneficial effect on the physical, social, and emotional well-being of dog owners, especially children and elderly people. The population of companion animals is increasing around the world. The number of dogs in Europe remains at a level of 2.9–8 million (UK: 9 m; France: 7.6 m; Poland: 7.3 m; Romania: 4.1 m; and Hungary: 2.9 m). In Brazil, the population of dogs is 35 million, and in the USA over 69 million [1]. Although companion animals play a significant role in human life, they may become a source of human-affecting pathogens including bacteria, fungi, viruses, and parasites. An example of zoonotic parasitoses is toxocarosis caused by larvae of *Toxocara* spp. [2,3,4,5]. Humans may also become infected with other non-specific parasites, for example, larvae of nematodes that belong to the family Ancylostomatidae [4]. Cases of infestation by whipworms *Trichuris vulpis* were described in children, although it remains debatable whether that invasion resulted from zoonosis [4,6].

Among the above-mentioned parasites, *Toxocara* spp. still poses the greatest risk for human health, especially in children [7]. These nematodes are frequently observed in puppies (*Toxocara canis*) and kittens (*Toxocara cati*). High fertility of nematodes and resistance of the eggs to environmental conditions, as well as the lack of prophylaxis and proper deworming schedules all result in systematic contamination of the environment with the eggs of parasites. *Toxocara* spp. is a cosmopolitan parasite commonly found in Poland. Data from the literature indicate that this parasite also creates frequent problems in several countries [8,9,10,11,12,13,14,15,16]. Apart from *Toxocara* spp., nematodes from the family Ancylostomatidae or Trichuridae are found in carnivorous animals. The source of infection for humans are invasive *Toxocara* spp. from the environment and migrant larvae which may cause two main clinical syndromes: ocular larva migrans (OLM) and visceral larva migrans (VLM). Additionally, in the environment there may be present invasive larvae from the Ancylostomatidae family which penetrate through the skin and lead to a disease called cutaneous larva migrans (CLM) [12,14,16,17]. The aim of the study was to determine the helminthic fauna of dogs originating from different areas and compare the findings with the results of previous studies. The authors also focused on the determination of the degree of contamination in an environment with zoonotic parasites.

## 2. Materials and Methods

### 2.1. Samples of Feces

In 2013, we examined the feces of 100 companion dogs of various breeds, 47 of which came from five districts of the city of Lublin (south-eastern Poland), and 53 dogs from five villages located 20 km away from Lublin. The age of dogs ranged from 2 months to 16 years. Six of the urban dogs were aged up to 1 year, 34 up to 2–5 years, and seven up to 6–10 years, while 11 of the rural dogs were aged up to 1 year, 21 up to 5 years, 18 up to 10 years, and three were over 10 years.

Fecal samples for the study were provided to the Department of Parasitology by pet owners according to instructions of veterinary practitioners.

Four-gram stool samples were examined macro- and microscopically. The macroscopic examination involved a careful inspection of the samples for visible parasites or their fragments. The microscopic examinations were carried out using a flotation technique involving a saturated solution of salt and sucrose (specific gravity 1.28–1.30) and a decantation method to evaluate qualitatively the composition of parasitofauna. As a quantitative method, we used the McMaster technique to determine the number of nematode eggs per 1 g of feces (EPG) [18]. Particular species were identified morphologically with Cell light microscope system software from Olympus [19].

### 2.2. Samples of Soil

A total of 35 samples of soil from sandpits, playgrounds, and home area pathways in urban and rural areas were examined (25 from Lublin and 10 from villages, respectively). The samples from Lublin were collected from five districts (15 sandboxes and 10 residential alleys). The samples from five villages were collected from 10 sandboxes (two sandboxes in each village). The samples were collected using the envelope method (from four corners and one from the center) from the surface layer and from layers located 30 cm underneath.

The soil samples were examined using the modified sedimentation-flotation method as described by Quinn et al. [20]. All soil samples (n = 35) examined by the Quinn method were investigated by means of LAMP (loop-mediated isothermal amplification). First, 1 g of sand or soil was taken from each sample to isolate DNA according to the method described by Borecka and Gawor [21]. Then, using a method that employs *Toxocara* DNA amplification in isothermal conditions (developed by Macuhova et al. [22]), the previously isolated DNA from the sand was tested using species-specific primers for the presence of genetic material from *T. canis* or *T. cati*. The amplified genetic material was separated by electrophoresis. A LED transilluminator was used to archive the results of molecular diagnostics.

### 2.3. Statistical Analysis

The statistical analysis of parasite occurrence depending on the environmental origin involved a chi-square test (including the results of the Z Fisher’s test with Bonferroni correction of the significant level) and two types of nominal correlation depending on the number of categories of variables (V Cramer and Phi). All analyses were performed using the PS Imago software package (IBM SPSS Statistics Version 23; SPSS Inc., Chicago, IL, USA). The chi-square test and Z Fisher’s test were used for precise determination of statistically significant differences between the presence of each parasite associated with an independent variable—the environment. Cramer’s phi correlation was used to demonstrate a relationship between the variables and its strength. A *p* value ≤ 0.05 was considered significant.

## 3. Results

### 3.1. Coproscopic Examinations

The total number of infected dogs was 38 (38%). The dogs infested with parasites were found in all of the surveyed city districts and villages. The prevalence in urban and rural dogs was 17.02% (n = 8) and 56.60% (n = 30), respectively. The percentage of dogs with zoonotic nematode eggs *(T. canis*, Ancylostomatidae, *T. vulpis*) was identical and amounted to 16% (Table 1).

Twenty-six dogs demonstrated single infection, mainly with *T. canis* and Ancylostomatidae. Co-invasions of two or three species of parasites (with hookworms and whipworms predominating) were reported in 12 dogs. An infection of four parasitic species (*T. canis,* Ancylostomatidae, *T. vulpis*, and *Capillaria aerophila*) was found in a four-year-old dog from a rural area.

The dogs from the urban areas revealed single infection. The highest number of eggs of zoonotic nematodes (*T. canis*, Ancylostomatidae or *T. vulpis*) were observed in the group of dogs aged ≤5 years. Only one dog (5 months old) had *T. canis* eggs. The dogs in this age group (up to 1 year old) were under constant veterinary supervision including vaccinations as well as regular and frequent deworming.

The dogs from rural areas demonstrated both single and co-infections. The largest number of dogs infected with *T. canis* were found in the group aged ≤1 year, with single invasions dominating. On the other hand, in the dogs from the group aged ≤5 years, Ancylostomatidae and *T. vulpis* infections dominated, with co-invasions observed most frequently.

The mean, minimum, and maximum number of nematode eggs per 1 g of feces (EPG) as well as the place of the dogs’ origin are shown in Table 2. Table 3 shows the number of dogs infected with zoonotic nematodes in the two environments in relation to the number of EPG (100, 101–500, 501–1000, or over 1000).

Analysis of parasitic occurrence in respect of the origin (the city or countryside), i.e., the independent variable, shows a significant correlation for all types of parasites (phi = −0.41, *p* < 0.001). The parasites were found more frequently (χ^2^ = 16, df = 1, *p* < 0.01) in the countryside (n = 30) than in city areas (n = 8). There is no correlation between environmental origin and the presence of *T. canis* (phi = −0.19, *p* > 0.05). However, a statistically significant correlation was found between the type of environmental origin and prevalence of Ancylostomatidae (phi = −0.30, *p* < 0.01). The parasite was found more frequently (χ^2^ = 9.10, df = 1, *p* < 0.01) in the countryside (n = 14) than in the city (n = 2). The analysis showed a significant correlation between the type of environmental origin and the occurrence of *T. vulpis* (phi = −0.60, *p* < 0.001). The parasite occurred more frequently (χ^2^ = 12.70, df = 1, *p* < 0.001) in villages (n = 15) than in cities (n = 1).

### 3.2. Results of Soil Examination

The results showed a high contamination rate of sandpits in urban (40%) and rural areas (60%), and residential alleys (70%). The highest number of parasite eggs was found in the layers at a depth of 30 cm. The examined sand samples revealed a few eggs, mainly *Toxocara* spp. in 100 g of sand. Only three samples had a higher number of eggs amounting from 11 to 20 (one urban and two rural sandpits). The residential alleys were similarly contaminated both on the surface and at deep layers.

Of the soil samples examined, 19 (54.3%) were positive for parasites eggs. The largest number of samples containing the eggs of *Toxocara* spp. were collected from home area pathways, both from the surface and deep layers (Table 4).

The analysis of parasite occurrence depending on the type of environment (sandpits in an urban area, residential area pathways in an urban area, and sandpits in a rural area) showed that there was no significant correlation (*p* > 0.05) between the type of environment and the general occurrence of two species of parasites (*Toxocara* spp. and Ancylostomatidae). There was no significant difference in the occurrence of parasites in pathways in an urban area (n = 7), sandpits in an urban area (n = 6), and sandpits in a rural area (n = 6). Also, there was no significant correlation between the deep layers examined and the overall occurrence of parasites (*p* > 0.05).

**Figure 1:**
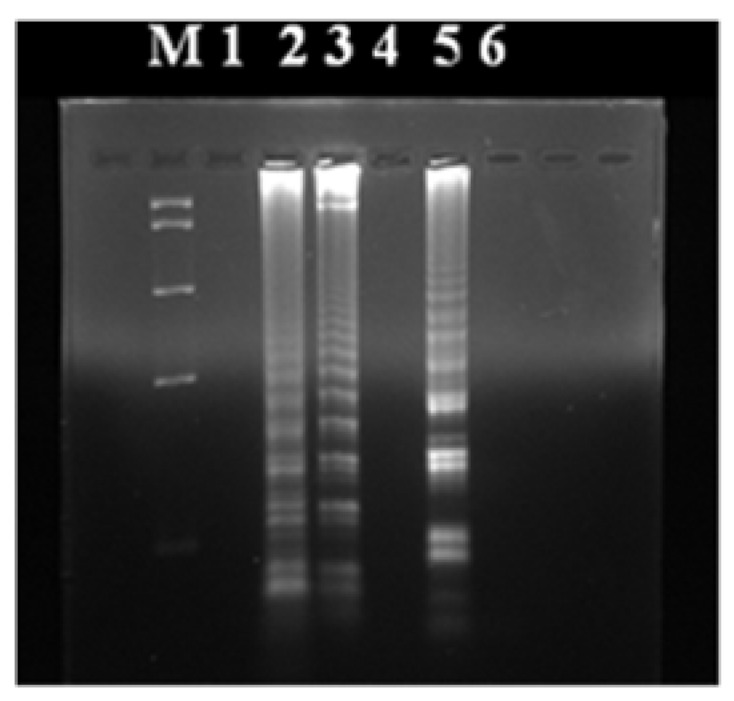
Effect of LAMP (loop-mediated isothermal amplification) reaction for *T. canis* and *T. cati*: Lane M—pUC Mix marker; Lanes 1 and 4—a negative control, Lane 2—a standard for *T. canis*, Lane 3—the product of reaction of LAMP DNA isolated from selected environmental samples (recovered from soil), Lane 5—standard for *T. cati*, Lane 6—no product of reaction of LAMP DNA isolated from the environmental samples.

The molecular examinations of sand and soil samples confirmed the presence of nematode eggs of the species *T. canis* in all sample classified previously as positive (Figure 1). Molecular studies ruled out the presence of genetic material from *Toxocara cati* in the tested environmental samples.

## 4. Discussion

Parasitological studies performed in various countries have indicated that canine infections are still a serious problem, subject to many factors, including the region of the country and the origin of animals. Moreover, the problem involves both homed and homeless dogs [3]. In some countries, the overall endoparasite prevalence remains at a level from a few to more than a dozen percent (9.36% in England, 5.9% in Finland, 8.1% in Holland, 9.4% in Germany, and 19.6% in Switzerland) [23,24,25,26,27]. However, in several countries the infection rate is much higher: 45.7% in Slovakia, 58.8% in Portugal, more than 50% in Hungary, and 75.5% in Serbia [8,10,11,13,28]. Our results (38%) also indicate a high percentage of dogs infected with parasites in the south-east of Poland. The prevalence varied markedly in different regions of Poland. Dogs from urban areas in central Poland revealed an infection rate of only 3.3% [29], whereas in north-western [30] and south-eastern Poland (our results) the prevalence was several times higher, amounting to 34.84% and 38.00%. A similar situation can be observed in north-western and southern Italy with a prevalence of 16% relative to a figure twice as high in central Italy [14,31,32]. It should be stressed that in addition to the origin of dogs, their environment may also affect the prevalence rate. Our results indicate that the positivity of dogs from rural areas is higher than that in urban areas (90.57% and 17.02%, respectively). Corresponding results were noted in the central region of Poland, where parasites were found more frequently in dogs from rural areas (34.2%) than from urban ones (3.3%) [29]. Similar results were found in the north-west region of Poland, with the prevalence rate amounting to 26.67–46.67% and 23.92%, respectively [30]. Other authors have also noted a higher prevalence in dogs from rural areas, for example, in Czech Republic the figure was 41.7% in the countryside and 17.6% in the city [33], and in Slovakia it was 66.0% and 39.1%, respectively [10].

Moreover, the degree of infection with zoonotic nematode eggs in dogs from rural areas was several times higher in 1 g of feces (the *T. canis* average EPG total was 853–1037 in the countryside, and 300 in the city). The evaluation of EPG indicates that 50% of all dogs examined were infected with 101 to 500 eggs/g of feces; of these animals only 10.5% came from urban areas. The dogs with 500 or more eggs in 1 g of feces (up to 3600 *T. canis* eggs and up to 4050 Ancylostomatidae eggs) also came from rural areas. This affects the degree of pollution with nematodes which is also crucial for human health. Considering the information obtained from dog owners, it may be concluded that a high number of EPG in rural areas is due to occasional and accidental dogs deworming. Similar results were found in The Netherlands and Portugal, where the average *T. canis* EPG was 889 [3] and 712.5, respectively [13]. However, in most other countries the number of eggs in 1 g of feces has reached the level of 100 [10,27,34].

In Europe, the percentage of dogs with zoonotic nematodes *T. canis* varies and remains at a rate from a few to several percent (7.1% in Switzerland, 5.3% in England, 4.6% in Holland, 3.1% in Finland, 6.26–13.7% in Czech Republic, 4.0% in Germany, 5.1% to 8% in Portugal, 12.4% in Denmark, 0.7–13% in central Italy, 16.5% in Slovakia) [13,23,25,26,28,33,35,36]. In comparison to western European countries, our results indicated a high proportion of dogs with *T. canis* (16%), where the majority of dogs with *T. canis* came from rural areas (75%). A similar situation is described in Czech Republic and in central Italy (the region of Marche), where prevalence found in dogs from rural areas was double the figure [33,37]. Considering our results from an earlier study in the same areas [38], there was a decline in the proportion of dogs with *T. canis* (from 34.9% to 16.0%) and this may be regarded as an important positive change. A similar situation may be observed in Germany, where the prevalence dropped from 22.4% to 4.0% [25,39]. Puppies and young animals are more likely to be infected with helminths than adult dogs. It should be emphasized that the data include primarily dogs from urban areas whereas infection in dogs from rural areas remains a serious problem [9].

A similar situation can be observed considering other zoonotic nematodes (Ancylostomatidae and *T. vulpis*). In Europe, the prevalence has also decreased, amounting to a few percent in recent years (respectively for the two parasites: 6.9% and 5.5% in Switzerland, 1.8% and 0% in England, 2.1% and 1.0% in The Netherlands , 2.6% and 0.2% in Finland, 0.8–1.6% and 1.1–1.7% in Czech Republic, 0.9% and 0.2–2.3% in Germany, 0.43–3.25% and 3.3–3.67% in Italy, 7.3% and 0% in Denmark, 2.5% and 1.5% in Slovakia) [14,23,24,25,26,27,32,33,35,36]. In contrast to other European countries, the prevalence of Ancylostomatidae and *T. vulpis* in rural areas of Portugal remains at a high level (40.9% and 29.9%, respectively) [13,28], while 18.4% and 10.0% in Slovakia [10]. Similarly, in southern Italy the *T. vulpis* prevalence rate reaches 10% [31] and in Hungary about 20.0% [8]. Our research shows that in south-eastern Poland the prevalence of Ancylostomatidae and *T. vulpis* is at a high level (16% and 16%, respectively), especially in dogs in rural areas (26.42% and 28.30%, respectively), and has not changed significantly for several years [38].

Zoonotic nematode parasites of dogs are cosmopolitan. Outside Europe, the percentage of dogs with *T. canis* also varies from a few to more than a dozen percent: 2.2% in the United States [40], 2.9% in Calgary (Canada) [41]. Similarly, the percentage of dogs infected with Ancylostomatidae and *T. vulpis* in these countries is low (2.5% and 1.2% in the United States [40], 0.81% and 0% in the region of Calgary (Canada) [41]). However, in less industrialized countries, the percentage of dogs infected by Ancylostomatidae and *T. vulpis* is much higher and varies from a few to more than ten percent. In Africa, the overall prevalence is high (62.6–82.5%), with hookworms dominating (35.3–53.8%), and the prevalence of *T. canis* and *T. vulpis* is much lower (3.3–15.8% and 3.7–7.9%, respectively) [15,42,43,44]. In Mexico, the prevalence is similar to that in Africa, with Ancylostomatidae also dominating (70.8%), and with the prevalence of *T. canis* and *T. vulpis* being 12.5% and 12.5%, respectively [34].

The rate of canine infection does not always reflect the pollution of the environment. Our results show that despite a decline in the percentage of dogs with *T. canis* (16.0%), the contamination of sand with nematode eggs is higher (45.7%). In other European countries (also in other regions of Poland), the pollution of the environment with parasites also poses a serious problem, and it varies depending on examined areas (parks, squares, playgrounds, sandpits, alleys in housing estates, etc.) [33,36]. In some areas, environmental pollution remains at a similar level in both urban and rural areas [44,45]. However, the data provided by several authors indicated that areas in cities are more contaminated than rural areas, 40.59% and 35.88%, respectively [46], 64.7% and 20.0% [47], and 11.9% and 5.0% [33]. Although our data indicate that pollution is higher in villages than cities (60% and 40%, respectively), similar results have been obtained from central Poland (30.4% and 23.3%, respectively) [48]. In Europe, the pollution of sandpits and parks has been studied the most frequently [32,35]. It is beneficial for humans that sandpits are often protected against the entry of animals and therefore either parasite-free or contain few parasite eggs [49,50]. In contrast, about 50% of unprotected sandpits in southern Poland may be contaminated [51]. Our findings indicate a high prevalence of pollution in sandpits with *T. canis* eggs in both urban and rural areas, amounting to 40%. This contrasts with southern regions of Poland, where only about 14% of sandpits were contaminated [52]. However, if we take into account alleys in housing estates found near sidewalks running in urban areas, we observe a high degree of contamination with *Toxocara* spp. (60%). In 2003, Tomczuk [46] obtained similar results in the same area (70.37%). It is obvious that this high prevalence rate resulted from feces that were not collected by dog owners [49]. Literature data indicate that despite the large number of contaminated sandpits, the quantity of eggs isolated from soil samples was small [48,50,53]. This fact is also confirmed by our results.

Most authors confirmed only the genus of *Toxocara* [33,36,37,45,46,47], so any discussion is rather difficult with respect to their results. Few authors who detected *Toxocara* spp. in the environment used molecular methods [54,55]. Ozlati [55] determined the genetic diversity from soils in public areas, identifying *T. canis* in 15.5%, *T. cati* in 27.2%, and mixed infections in 12.2%. Similarly, Khademvatan [53] identified *T. cati* more often than *T. canis* (28% and 5.7% respectively), and no mixed contamination was observed. In contrast to other authors, our molecular research demonstrated only *T. canis* (45.71%).

Dogs with parasites affect environmental contamination, which is the main source of human infections with zoonotic nematodes. Notably, dogs from rural areas are more affected by *T. canis* than those from urban areas [37,48]. Toxocarosis is one of the most common zoonotic helminth infections worldwide. The source of infection of humans is believed to be invasive eggs of *Toxocara* spp. from the environment [2,3,5,7,16,34,48]. The main cause is *T. canis*, which is considered a serious zoonosis and may cause ocular larva migrans (OLM) or visceral larva migrans (VLM) or not manifest clinical symptoms at all. Lately, *T. cati* is recognized by experts to be a zoonosis that can cause VLM and OLM [16].

The seropositivity in humans from different countries ranges from several to several tens of percent. In Western countries, it was found that 2% to 5% of apparently healthy adults from urban areas were seropositive compared to 14.2% to 37% of adults in rural areas [2]. In The Netherlands, anti-*Toxocara* spp. antibodies were higher in people older than 45 years old (30%) relative to those younger than 30 years old (4–15%) [3]. In Mexico, however, the serological prevalence of *T. canis* in humans from a rural community was 29.2% and at a lower level in people under 40 years of age (27.6%) [34]. In Poland, in the period between 2002 and 2005, for children suspected of being infected with *Toxocara* spp. the seropositivity was about 75.6% [5,56].

Moreover, it should be stressed that the percentage of infected people in Poland has been on the rise in recent years [7,57,58]. In 2005, the percentage of seropositive people was 76% in central areas of Poland [5]. The results indicate that despite campaigns on risks posed by carnivores and the need for prevention, dog owners show no sense of responsibility. A lack of regular deworming and parasitic examinations leads to continuous environmental pollution. Moreover, sandpits without fences contribute to a higher risk to human health.

## 5. Conclusions

In conclusion, this study revealed that 38% dogs had parasitic infections. A higher prevalence was observed in rural areas (56.60%) in comparison to urban areas (17.02%). The percentage values of nematodes important for human health (*Toxocara canis*, Ancylostomatidae, *Trichuris vulpis*) were similar (16%). The infected dogs were dominated by single species, with the main species being *T. canis* (28.95%). Regarding sandpits, higher contamination rates were observed in rural (60%) than urban (40%) areas. In total, 54.30% of the soil samples were contaminated with parasite eggs, mainly *Toxocara* spp. The molecular examinations (LAMP method) of soil samples confirmed the presence of nematode eggs of the species *T. canis* in all samples previously classified as positive. Similar to other countries, in Poland, despite campaigns on the risks posed by carnivores and the need for regular deworming, the greatest parasitic threat to people is from the contamination of the environment.

## Figures and Tables

**Table 1:** Prevalence of parasites in dogs.

Number of Dogs Examined		Number of Dogs Infected				
Total	Infested	*Toxocara* *canis*	*Toxascaris* *leonina*	*Ancylostoma-* *tidae*	*Trichuris* *vulpis*	*Capillaria* *aerophila*
100	38 (38%)	16 (16%)	3 (3%)	16 (16%)	16 (16%)	5 (5%)

**Table 2:** Eggs per 1 g of feces (EPG) in canine feces from various environments.

	No. of Dogs Infested	EPG Mean (Min–Max)	Urban Area		Rural Area	
			No. of Dogs Infested	EPG Mean (Min–Max)	No. of Dogs Infested	EPG Mean (Min–Max)
*T. canis*	16	852.81 (50–3600)	4	300 (100–550)	12	1037 (50–3600)
*T. leonina*	3	383.33 (50–850)	1	50	2	500 (150–850)
*Ancylosto-matidae*	16	584.38 (50–4050)	2	700 (50–1350)	14	568 (50–4050)
*T. vulpis*	16	134.38 (50–300)	1	50	15	137 (50–300)
*C. aerophila*	5	60 (50–100)	0	0	5	60 (50–100)

**Table 3:** The number of infected dogs from various environments with different levels of EPG.

EPG	*Toxocara canis*			Ancylostomatidae			*Trichuris vulpis*		
	Total	Urban	Rural	Total	Urban	Rural	Total	Urban	Rural
<100	2	1	1	5	1	4	8	1	7
101–500	7	2	5	8	0	8	8	0	8
501–1000	3	1	2	1	0	1	0	0	0
>1000	4	0	4	2	1	1	0	0	0

**Table 4:** The presence of eggs of intestinal parasites in sandpits in the city of Lublin and rural areas.

Sampling Site			Total	No of Positive Samples (%)	No. of Invasions		
					Single		Mixed
					T	A	T/A
urban area	sandpits	surface	15	2 (13.3)	2	0	0
		depth 30 cm		4 (26.7)	4	0	0
	home zone pathways	surface	10	0	0	0	0
		depth 30 cm		1	0	1	0
		surface + depth		6	3	0	3
rural areas	sandpits	surface	10	1 (10)	1	0	0
		depth 30 cm		5 (50)	3	2	0

T—*Toxocara canis*, A—Ancylostomatidae, T/A—*Toxocara canis*/Ancylostomatidae.
